# Using ensembles and distillation to optimize the deployment of deep learning models for the classification of electronic cancer pathology reports

**DOI:** 10.1093/jamiaopen/ooac075

**Published:** 2022-09-13

**Authors:** Kevin De Angeli, Shang Gao, Andrew Blanchard, Eric B Durbin, Xiao-Cheng Wu, Antoinette Stroup, Jennifer Doherty, Stephen M Schwartz, Charles Wiggins, Linda Coyle, Lynne Penberthy, Georgia Tourassi, Hong-Jun Yoon

**Affiliations:** Oak Ridge National Laboratory, Oak Ridge, Tennessee, USA; University of Tennessee, Knoxville, Tennessee, USA; Oak Ridge National Laboratory, Oak Ridge, Tennessee, USA; Oak Ridge National Laboratory, Oak Ridge, Tennessee, USA; College of Medicine, University of Kentucky, Lexington, Kentucky, USA; Louisiana Tumor Registry, Louisiana State University Health Sciences Center School of Public Health, New Orleans, Louisiana, USA; Rutgers Cancer Institute of New Jersey, New Brunswick, New Jersey, USA; Utah Cancer Registry, Huntsman Cancer Institute, University of Utah, Salt Lake City, Utah, USA; Fred Hutchinson Cancer Center, Epidemiology Program, Seattle, Washington, USA; University of New Mexico, Albuquerque, New Mexico, USA; Information Management Services Inc., Calverton, Maryland, USA; National Cancer Institute, Bethesda, Maryland, USA; Oak Ridge National Laboratory, Oak Ridge, Tennessee, USA; Oak Ridge National Laboratory, Oak Ridge, Tennessee, USA

**Keywords:** ensemble distillation, CNN, NLP, deep learning, selective classification

## Abstract

**Objective:**

We aim to reduce overfitting and model overconfidence by distilling the knowledge of an ensemble of deep learning models into a single model for the classification of cancer pathology reports.

**Materials and Methods:**

We consider the text classification problem that involves 5 individual tasks. The baseline model consists of a multitask convolutional neural network (MtCNN), and the implemented ensemble (teacher) consists of 1000 MtCNNs. We performed knowledge transfer by training a single model (student) with soft labels derived through the aggregation of ensemble predictions. We evaluate performance based on accuracy and abstention rates by using softmax thresholding.

**Results:**

The student model outperforms the baseline MtCNN in terms of abstention rates and accuracy, thereby allowing the model to be used with a larger volume of documents when deployed. The highest boost was observed for subsite and histology, for which the student model classified an additional 1.81% reports for subsite and 3.33% reports for histology.

**Discussion:**

Ensemble predictions provide a useful strategy for quantifying the uncertainty inherent in labeled data and thereby enable the construction of soft labels with estimated probabilities for multiple classes for a given document. Training models with the derived soft labels reduce model confidence in difficult-to-classify documents, thereby leading to a reduction in the number of highly confident wrong predictions.

**Conclusions:**

Ensemble model distillation is a simple tool to reduce model overconfidence in problems with extreme class imbalance and noisy datasets. These methods can facilitate the deployment of deep learning models in high-risk domains with low computational resources where minimizing inference time is required.

## BACKGROUND AND SIGNIFICANCE

The American Cancer Society (ACS) estimates 1.9 million new cancer cases will be diagnosed in 2022.^1^ Because cancer is a reportable disease, states rely on population-based registries to maintain a database of cancer pathology reports. Information contained in these documents is key to identifying new reportable cancers and their characteristics across the country.

Electronic pathology reports are stored as unstructured text, and current information extraction relies almost completely on manual processing by trained personnel, which is expensive, time-consuming, and prone to error. In the last few years, researchers have reported promising results when training deep learning (DL) models to automate the information-extraction process for pathology reports.^2–5^

Data noise is a serious issue when training DL models for classifying cancer pathology reports. Documents often describe multiple specimens and biopsies that involve different organs analyzed for diagnosis. Manual annotators read the results of each biopsy and assign a specific cancer site label for the entire report. Although this is a standard way to annotate data, this process leads to a large volume of data noise because large portions of pathology reports focus on the analysis of specimens that are associated with a different site and are not relevant to the context of their ground-truth label. Pathology reports also include information (eg, names and addresses) that contributes to additional noise. Training neural networks with noisy data can yield models that learn spurious correlations and shortcuts.^6^^,^^7^

Label noise presents additional challenges. Annotators are tasked with selecting a class out of hundreds of options. Tasks such as *cancer subsite* and *histology determination* involve the identification of specific classes that often share similarities (eg, “overlapping lesion of other and unspecified parts of mouth,” “mouth not-specified”). Human annotation errors will naturally occur when working with a large number of similar classes and documents in which multiple specimens associated with different classes are reviewed. In addition, errors can derive from data processing. For example, labels are defined at the cancer/tumor/case (CTC) level. CTC is a data entity that encapsulates all diagnostic, staging, and treatment for a reportable neoplasm. Consequently, pathology reports created during diagnosis are assigned labels based on the CTC—even if these documents analyze specimens associated with different labels.

Extreme class imbalance combined with data noise can lead to serious overfitting issues. The cancer subsite and histology coding tasks consist of 326 and 639 classes, respectively. Some of the classes in these tasks are extremely common. For example, in the subsite task for breast cancer, *upper-outer quadrant of breast* constitutes 8.9% of the data, whereas for the top class in histology, *adenocarcinoma, NOS* corresponds to 21.7% of the data. On the other side of the spectrum, there are cancer types that rarely appear. There are 16 classes in subsite and 127 classes in histology with less than 10 instances. When few samples are available during training, DL models tend to memorize specific patterns that do not generalize well.^8^^,^^9^ Thus, overfitting is a major challenge when classifying cancer pathology reports.

During classification, it is often desirable to keep only the predictions produced with high confidence. For example, cancer registries from Kentucky, Utah, New Jersey, Washington, and New Mexico (Section III-A) currently require machine learning models to achieve 97% accuracy on a standard test dataset before deployment. The high accuracy imposed on models is necessary to limit potentially costly mistakes in processing healthcare records. Like other high-risk fields such as self-driving cars and medical diagnosis, the goal is to minimize error and maximize coverage. Previous investigators have referred to this research area as *selective classification*,^10–12^*prediction with a reject option*,^13^ and *model abstention.*^14^

The literature on selective classification for traditional machine learning is extensive, with one of the first papers published in the 1950s.^13^^,^^15^^,^^16^ However, few papers have discussed model abstention in the context of DL.^10^ Previous work in this area focused mostly on deriving an optimal softmax threshold given a certain cost/risk constraint. Because softmax layers are common in DL architectures, the softmax thresholding framework is a simple and convenient rejection rule that can be applied to most models, including pretrained networks.

Model overconfidence deteriorates the efficiency of abstention mechanisms that are based on softmax thresholding. Previous researchers have hypothesized about the source of model overconfidence. They pointed out that using one-hot (hard) labels during training leads to overconfidence because it encourages the model to produce predictions with 100% confidence.^17^^,^^18^ From an overfitting perspective, overconfidence is the result of overfitting the negative log-likelihood loss, which encourages the model to produce outputs with low entropy.^19^ In addition, hard labels do not allow for degrees of truth, and assigning 100% confidence to noisy documents that mention specimens associated with numerous classes may not accurately represent the input. Model overconfidence leads to a larger volume of highly confident but wrong predictions, and that has a direct negative impact in abstention mechanisms that are based on softmax thresholding.

One could train models with soft labels to reduce overconfidence and provide accurate input representation. However, creating accurate soft labels imposes several challenges. Manual creation of soft labels would involve assigning probabilities to each class, which can be subject to the annotator’s interpretation and is often not feasible owing to time constraints.

A simple way to derive soft labels is label smoothing. Given some constant, *α*, with α∈(0,1), this method assigns a 1−α to the ground-truth class and splits *α* equally among the rest of the classes (ie, 0 becomes $\frac{\alpha }{K-1}$, where *K* is the number of classes). However, label smoothing does not introduce any information about the underlying class hierarchies or knowledge related to the quality of the input. In addition, although label smoothing can potentially reduce model overconfidence that does not imply improved abstention performance when choosing a softmax threshold. In fact, we hypothesize that this method is likely to deteriorate abstention rates because it is prone to shorten the distribution of model predictions (ie, from [0,1] to [0,α]), thereby making it more difficult to find a softmax threshold that separates between right and wrong predictions.

Ensemble learning is a simple solution to reduce overfitting. The benefits of ensembles in the context of overfitting have been quantified extensively by previous researchers.^20^^,^^21^ Highly parallelizable ensemble methods are especially attractive because they can be implemented and tested quickly. However, cancer registries across the country have limited computing resources, thereby making ensemble methods unfeasible in the deployment/inference phase. Ensembles also require additional testing time because a single prediction often requires the output of every single model in the ensemble. Thus, ensembles remain a computationally expensive technique and that limits their utility and prevents their deployment in numerous environments.

Model distillation is a promising, low-resource solution that leverages the benefits of ensembles without using hard labels. The idea behind model distillation is to transfer and compress the knowledge of a larger model (teacher) into a smaller (student) network. In the context of ensemble model distillation, researchers have attempted to transfer the combined knowledge of a group of models into a single, low-resource network.^22^ Thus, they aim to maintain the high performance of the ensemble while enjoying the computational flexibility of a single model. One intuitive way to perform ensemble model distillation is to train a student model with soft labels obtained through the aggregation of the ensemble predictions. That is, training the student model using ensemble predictions instead of the annotated labels. This method permits automatic derivation of soft labels that contain information about the variability within the ensemble and avoids the use of hard labels.

Previous work explored model abstention for ensemble learning.^23^^,^^24^ Existing work focused on deriving rejection boundaries based on the statistics of the ensemble predictions. The downside of these studies is that they focused on simple binary problems, and more complex classification tasks, such as the ones we have described for electronic pathology report information extraction, are not considered. To the best of our knowledge, the effect of ensemble model distillation in the context of selective classification remains an understudied research area.

## OBJECTIVE

The objective of this study was to investigate the feasibility of ensemble model distillation as a low-resource alternative for the deployment of DL models for cancer pathology report classification. We hypothesized that ensemble model distillation would allow us to enjoy both the overfitting reduction benefits of the ensemble and a reduction of model overconfidence caused by hard labels. Performance was quantified as accuracy and abstention rates by using softmax thresholding tuned to yield 97% accuracy. We provided additional analysis of the benefits of ensemble model distillation on data and label noise. These findings may provide solutions to other machine learning researchers working in high-risk domains with limited computational resources where low-error rates are required.

## MATERIALS AND METHODS

### Dataset

Classifying electronic cancer pathology reports consists of 5 individual tasks. That is, each pathology report must be labeled with a specific site, subsite, laterality, histology, and behavior. The number of classes in each task is shown in Table 1.

**Table 1.** Number of classes in each task

Task	Site	Subsite	Laterality	Histology	Behavior
Classes	70	326	7	639	4

For this study, we used datasets from the Louisiana Tumor Registry (LTR), Kentucky Cancer Registry (KCR), Utah Cancer Registry (UCR), New Jersey State Cancer Registry (NJSCR), Seattle Cancer Registry (SCR), and New Mexico Tumor Registry (NMTR). The sizes of the 6 individual datasets are listed in Table 2. To satisfy deidentification requirements, we used integers instead of the actual names to represent each of the datasets.

**Table 2.** Size of individual registries

Registry	R1	R2	R3	R4	R5	R6
e-Path Reports	85 789	577 094	137 135	441 732	360 375	365 152

The dataset exhibits extreme class imbalance. For example, the top 2 histology classes (ie, *adenocarcinoma*, *NOS* and *ductal carcinoma*) constitute 41.0% of the dataset. In the subsite task, the top 2 classes (ie, *upper-outer quadrant of breast* and *prostate gland*) correspond to 17.3% of the data. It is not uncommon to see fewer than 10 instances for rare cancer types.

### Experimental setup

We aimed to develop experiments that would simulate real-world deployment. To achieve this, we implemented a leave-one-registry-out approach in which we first combined 5 registries for training and validation. Once the model was trained, we deployed the model by predicting the left-out (out-of-distribution) dataset. We expected the left-out dataset to contain natural variations not observed during training.

This experimental setup simulates real-world deployment and allows us to evaluate the generalizability of the classifier. In a previous study,^25^ we quantified the performance disparity between a test dataset, which was taken from the same distribution as the training and validation data, and a completely unseen (left-out) registry. In that study, we observed that R4 exhibited the largest performance drop. Therefore, for this study, we used R4 as our left-out dataset. Leaving out R4 and combining the rest of the registries leads to a total of 1 525 545 pathology reports for training and validation and 441 732 (size of R4) documents for testing.

**Figure 1.**
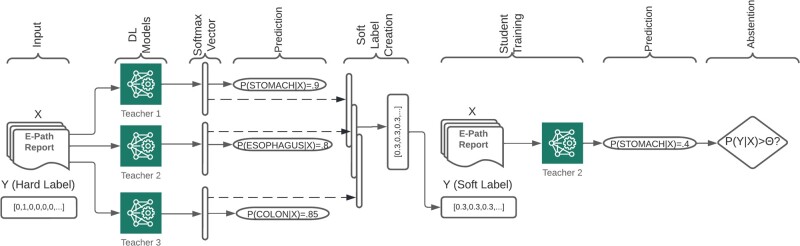
Overview of our training pipeline with a hypothetical example in which 3 different models classify a pathology report as stomach, esophagus, and colon. Our actual implementation consists of 1000 teacher models.

DL models were trained using early stopping as a standard overfitting prevention practice. We set the patience parameter to 5, so if the validation loss does not decrease for 5 consecutive epochs, then training stops, and the model recovers the best set of weights. Figure 1 shows an overview of our training pipeline.

### Multitask TextCNN

The base model for our experiments is the TextCNN. We use this specific DL model because: (1) in our previous publication involving pathology report classification, we showed that the TextCNN model performs about the same or better than transformer-based models,^5^ (2) we can train ensembles of multitask convolutional neural networks (MtCNNs) in parallel since it is a computationally cheap model (in terms of memory and speed), and (3) the low computational requirements of the MtCNN makes it accessible to cancer registries across the country, allowing for rapid deployment.

We used a specific version of the TextCNN known as the MtCNN, which has been implemented in numerous previous studies involving cancer pathology report classification.^2^^,^^4^^,^^25–28^ The MtCNN simultaneously outputs predictions for all 5 tasks. The input to the MtCNN first passes through an embedding layer, in which each word token is mapped to a 300-dimensional word-embedding vector. The resulting matrix passes through 3 parallel convolutional layers with filter sizes of 3, 4, and 5 consecutive words; each of the convolutional layers contains 300 filters. The output of the convolutional layers is then concatenated and sent to a global max pooling over time layer. Finally, the resulting vector goes through 5 parallel dense layers (one for each of the 5 classification tasks), and the 5 predictions are produced.

### Ensemble learning

Ensemble learning is a machine learning method that utilizes multiple models to obtain better predictions. Several ensemble learning algorithms are available, including bootstrap aggregation (bagging),^29^ boosting,^30^ and a mixture of experts.^31^

Previous studies have noted that training deep neural networks with more data led to better performance, and bagging can hurt performance because models only see ∼63% of the data.^32^^,^^33^ These studies showed that using the entire training dataset is more efficient than bagging approaches, which sample the training dataset with replacement. Therefore, in this study, we trained 1000 MtCNN models using the entire training dataset but different random initialization seeds. Our method is highly parallelizable and simple to implement.

The ensemble inference was derived by normalizing the summation of the outputs from the multiple models: $D(x)=\sum_{i=1}^{T}d_{i}(x)$, where **x** is some document input, *d_i_* is the prediction vector for model *i* in the ensemble, and *T* is ensemble size (*T *=* *1000). Then we applied the softmax function to the ensemble output *D* to infer the ensemble decision.

Notably, to obtain a prediction for a given document, *x*, one must first use all *T* models for the prediction and then aggregate their predictions to obtain the final output. This can be time-consuming and computationally demanding. Therefore, ensemble learning is often a nonviable method for several real-world applications.

### Distillation

In a typical supervised learning setting, a neural network is trained with data in the form (*x*, *y*), where in our case, *x* is a pathology report, and *y* is the associated label depending on the task (eg, cancer site, subsite). This type of learning uses *hard labels*, which means *y* is a one-hot encoded vector that contains binary information: either *y* belongs to a certain class or it does not (ie, [0,0,1]). Alternatively, one could train a model with data in the form of $\left(x,\vec{y}\right)$, where $\vec{y}$ could be interpreted as the probability that *x* belongs to each class (ie, [0.1,0.1,0.8]). This paradigm is known as *soft labeling*.

To distill the knowledge of the ensemble, we trained a single MtCNN using the ensemble predictions (aggregated vectors) as the class labels for the respective documents. Thus, the student model was trained with the same training documents, *X*, but with the soft labels derived from the ensemble instead of the original hard labels. Notably, our distillation implementation uses the categorical cross-entropy loss function: $CE(y,\hat{y})=\sum_{k=1}^{K}y*\;\text{log}\;\hat{y}$ where *y* is the soft label, $\hat{y}$ if the softmax prediction vector, and *K* is the number of classes. The following list describes the steps we took to distill the knowledge of the ensemble model:

- Train 1000 MtCNN models.
- Extract the softmax prediction vector of the 1000 MtCNNs for each document in the training set.
- Aggregate the prediction vectors by summing and normalizing. This will form a new set of $\vec{y}$ labels, where the labels are vectors (soft labels).
- Train a single MtCNN using the original pathology reports, *X*, but with the soft labels $\vec{y}$ instead of the original ground-truth labels.

By using the soft labels of the ensemble as the truth labels, we hypothesized that the model would identify the same features that the ensemble used to produce the classification probabilities.

### Selective classification with softmax thresholding

In numerous applications such as ours, low error rates are tolerated. In these problems, abstention mechanisms are implemented so that only models with highly confident predictions are used. In this study, we seek to maintain an error rate below 3% based on the consensus of state cancer registry partners.

To accomplish this, we implemented a straightforward version of model abstention that can be used with any trained model. Our goal was to satisfy the requirements by finding a softmax threshold that will yield 97% accuracy. The specific procedure is described as follows:

- Create a list of potential thresholds from 0 to 1 with a step size of 0.001 (ie, [0.001, 0.002, …, 0.999]).
- Using the validation dataset, find the smallest threshold so that when predictions are filtered out with a softmax below this threshold, the accuracy is ≥0.97.
- Use this threshold as a rejection rule. At test time, discard predictions that have a confidence below the selected threshold.
- Compute the percentage of documents that remain in the dataset after abstention and measure accuracy of that subset of the data.

Although the abstention rate refers to the proportion of documents left out during testing, in this study, we report results in terms of *retention proportions*: $RP=\frac{\hat{X}}{X}$ where $\hat{X}$ is the number of predictions made with confidence higher than the rejection threshold, and *X* is the size of the dataset. Also note that RP = (100% − abstention rate). Ideally, one will retain a large percentage of the data (high coverage) and obtain an accuracy of ∼0.97 on these documents.

### Statistical significance

We wanted to analyze if the student model performance was statistically better than a standard MtCNN model trained with hard labels. To account for natural variation, we used the 1000 MtCNN models that we trained for the ensemble to derive 95% confidence intervals.

### Model overconfidence

We analyzed model overconfidence by focusing on wrong predictions. We first determined the distribution of prediction confidence (softmaxes) for all wrong predictions. In this analysis, we compared the baseline MtCNN with the student model to understand the extent to which the student model can reduce the number of wrong predictions with high confidence.

In our second analysis of model overconfidence, we analyzed the number of highly confident but wrong predictions for different ensemble sizes. In particular, we quantified the number of wrong predictions made with a confidence of >0.97. Our choice of a 0.97 threshold is based on the consensus of the cancer registries’ error tolerance. This analysis was performed by averaging multiple samples obtained by bootstrapping MtCNNs from the pool of 1000 models and then creating ensembles of the respective sizes.

### Data and label noise analysis

We hypothesized that data and label noise were both issues when training models for classifying cancer pathology reports. We investigated the effects of model distillation on data and label noise by examining the distribution of ensemble predictions and inspecting individual reports.

We first verified that the ensemble predictions could effectively fix noisy labels. To that end, we inspected documents in which a wrong prediction was made with 100% agreement between the 1000 models.

Our analysis of data noise was based on the assumption that the amount of noise contained in the input will manifest itself in the distribution of votes across the ensemble. Traditional majority voting uses Equation 1 to infer the ensemble predictions,^34^ where $d_{t,j}=1$ if model *t* of the ensemble *T* predicts class *j* and $d_{t,j}=0$ otherwise. Here, we were interested in cases where the number of votes was split almost equally between 2 or 3 classes, meaning that there is not a clear winner. For cases in which half of the ensemble predicts class *y*_1_, and the other half predicts *y*_2_, we expected to observe a report that contained lexical patterns common to both classes.

(1) $$\underset{1\leq j\leq k}{\max}\sum_{t=1}^{T}d_{t,j}.$$

The distribution of ensemble votes has a direct impact on the derived soft labels. For example, given a pathology report about the gum, half of the ensemble may predict *upper gum*, and the other half may predict *lower gum*. Naturally, the resulting soft label for such input is expected to represent such a division (ie, [0.5,0.5,0, …] = [*lower gum*, *upper gum*, …]). Thus, we wanted to visualize what aspect of the input drives this type of predictive pattern and how that relates to the derived soft label.

## RESULTS

### Selective classification

Table 3 presents the results in terms of retention proportions (ie, the percentage of documents that would be classified when deploying the models). As expected, when combining predictions from multiple classifiers, the ensemble model yielded the best overall performance. We also observed that the student model outperformed the baseline MtCNN for all tasks except for behavior (the task containing only 4 classes). We note that the major boost in performance was observed for subsite and histology, for which the absolute increase in coverage was ∼1.81% and ∼3.33%, respectively. These are the 2 most difficult tasks because they are characterized by a large number of classes and severe class imbalance.

**Table 3.** Retention proportions results

Model	Site	Subsite	Laterality	Histology	Behavior
MtCNN	90.62	34.52	87.83	23.87	99.46
	(90.61, 90.63)	(34.48, 34.56)	(87.82, 87.85)	(23.77, 23.97)	(99.45, 99.46)
Student	91.10	36.33	88.49	27.20	99.98
Ensemble	92.17	39.00	89.60	34.16	99.42

*Notes*: The numbers shown represent the percentage of document remaining after abstention (higher percentage means more coverage). Intervals represent 95% confidence intervals.

MtCNN: multitask convolutional neural network.

Using the percentages from Table 3 and the size of the test dataset (R4: 441 732), one can translate these values into the number of pathology reports. For example, in subsite, the MtCNN and the student model classify 152 485 and 160 481 of the 441 732 reports, respectively. This indicates that the student model can be used to classify an additional 7996 documents. A similar calculation for the histology task showed that the student model can predict an additional 14 710 pathology reports (ie, 120 151 − 105 441 = 14 710).

Table 4 lists the accuracy scores obtained among the nonabstained documents. Although the individual softmax thresholds were tuned with the validation dataset to yield a 97% accuracy, we still observed accuracy below our target performance in all tasks except for behavior. This type of drop was expected owing to the natural distribution shifts when applying models to new registries, and in practice, this drop can be easily mitigated by using a higher target accuracy. Notably, the baseline MtCNN exhibits performance well below the 97% target (see subsite and histology in Table 4). The student model alleviates the performance drop, but it still fails to reach the target.

**Table 4.** Accuracy results

Model	Site	Subsite	Laterality	Histology	Behavior
MtCNN	96.06	94.43	96.05	95.12	97.69
	(96.06, 96.07)	(94.42, 94.45)	(96.04, 96.05)	(95.10, 95.14)	(97.69, 97.70)
Student	96.27	94.82	96.19	95.84	97.60
Ensemble	96.19	94.55	96.10	95.78	98.00

*Note*: Intervals represent 95% bootstrap confidence intervals.

MtCNN: multitask convolutional neural network.

### Wrong prediction confidence

**Figure 2.**
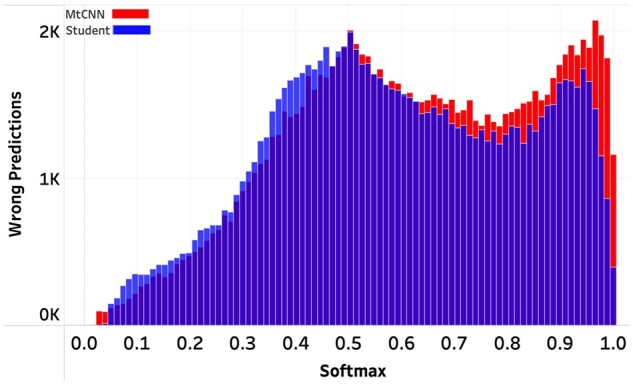
Histology Task. Distribution of softmaxes for the wrong predictions.

**Figure 3.**
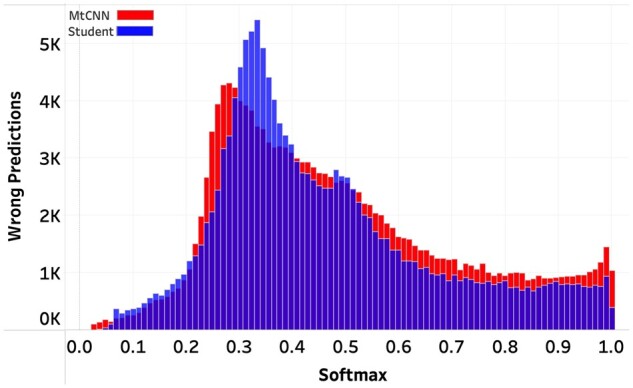
Subsite Task. Distribution of softmaxes for the wrong predictions.

When implementing a softmax-threshold abstention mechanism, minimizing the number of highly confident but wrong predictions is essential. That is because having too many highly confident wrong predictions pushes the softmax threshold toward 1, thereby leading to higher abstention percentages (ie, less coverage). We compared the prediction confidence distributions of the wrong predictions for histology (Figure 2) and subsite (Figure 3). For both tasks, the student model generated fewer wrong predictions with softmaxes above 90%, and this difference was particularly noticeable in the histology task. These plots illustrate the effects of training models with hard and soft labels and their impact on selective classification scores.

**Figure 4.**
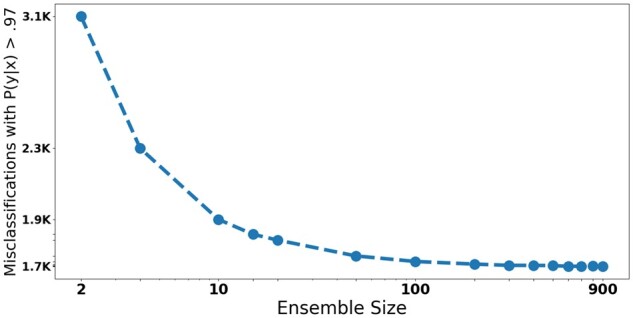
Wrong histology predictions made with confidence >0.97.

Figure 4 shows the effects of ensemble sizes on the number of wrong predictions made with a confidence >0.97. We observed that the number of wrong predictions decreased as the ensemble sizes increased, but this trend converges after an ensemble size of approximately 200 models. We also note that most of the improvement occurs with the addition of the first few models. That is, the ensembles with between 2 and 10 models exhibit the highest performance boost.

### Data and label noise analysis

**Figure 5.**
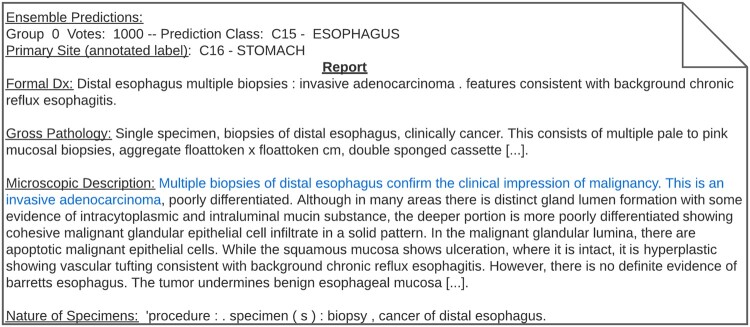
Incorrectly annotated pathology that was fixed during the distillation process. Some sentences were removed to conserve privacy.

We analyzed the effects of ensemble model distillation in terms of label noise by manually reading pathology reports that were classified incorrectly with 100% ensemble agreement. In every document we inspected, we found that those documents were mistakenly annotated. As an example, we deidentified one of the documents that was annotated as *stomach* but was classified as *esophagus* by every model (Figure 5). This was a case in which a pathology report discussed the biopsy of only one specimen, and lexical patterns tend to be consistent with the predicted class. Notably, in cases in which there is a 100% agreement within the ensemble, the associated softmax approximates the hard label (eg, the predicted class contains a value close to 1).

**Figure 6.**
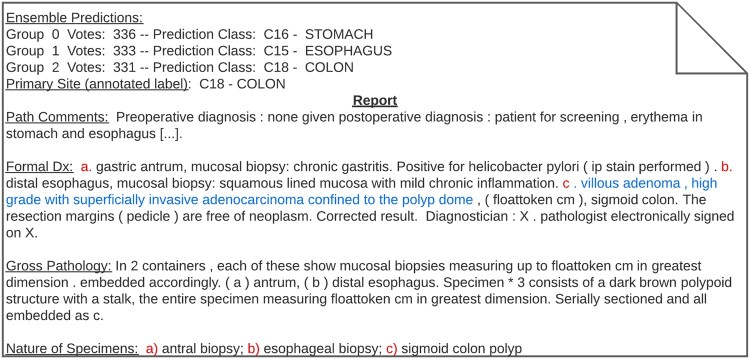
Pathology report in which the ensemble prediction votes were split into 3 equivalent groups. This report includes results of 3 analyzed specimens related to the stomach, esophagus, and colon. Some sentences were removed to ensure privacy.

We also analyzed data noise by examining individual pathology reports based on particular ensemble prediction patterns. Intuitively, we expected that when the ensemble votes were split across multiple sites, the pathology report would discuss specimens and biopsies that were associated with each of the predicted sites. As an example, we deidentified a pathology report in which the ensemble votes were split into 3 equivalent-size groups for the predictions of *stomach*, *esophagus*, and *colon* (Figure 6). This is an example where the input contained lexical patterns related to all 3 classes (see sections *path comments* and *nature of specimens* in Figure 6), and this misled the models’ predictions.

## DISCUSSION

This is the first study to quantify the abstention performance of ensemble model distillation by using softmax thresholding for the classification of electronic cancer pathology reports. Our results indicate that soft labels derived through ensemble model distillation can effectively improve abstention performance. Therefore, in real-world settings where inference resources are limited, our proposed distillation method yielded considerable improvements while maintaining the computational cost of a single model.

We measured performance by calculating the percentage of documents kept in the dataset after abstention and the accuracy of those documents. We observed that the student model outperformed the baseline MtCNN under both metrics and for 4 of the 5 tasks. The most substantial improvements occurred in the 2 most difficult tasks (subsite and histology), which contained the highest number of classes (326 for subsite and 639 for histology). Thus, we showed that ensemble model distillation can help increase the DL models’ coverage when deploying models in the real world, thereby allowing practitioners to use AI systems in a larger volume of documents and reduce the cost and time associated with manual annotation.

Although the performance increase obtained with the student model may seem small, these improvements become substantial at a large scale. For the subsite and histology tasks, the student model increases coverage by ∼1.81% and ∼3.33%, respectively. These values represent 7996 and 14 710 pathology reports using our current test dataset. However, given the ACS predicts that 1.9 million new cancer cases could be diagnosed in 2022,^1^ and it takes ∼2 min to read each report, these percentages could represent 10 000+ pathology reports and 1000s of hours of manual annotation saved.

Analyzing the distribution of ensemble votes and their resulting soft labels yielded additional insight into data noise in pathology reports. During cancer diagnosis, it is common to analyze multiple samples from tissues outside of the diseased organ. The result is multiple pathology reports from numerous biopsies. We argue that data noise is a serious issue in these reports because they contain lexical patterns commonly associated with classes outside of their assigned ground-truth label. When analyzing the ensemble vote distribution, we found that these particularly confusing pathology reports can be identified by inspecting documents in which the ensemble votes are split into equivalent-sized groups (Figure 6). In these cases, assigning a soft label allows for degrees of truth to indicate that the word patterns and context of the input do not belong exclusively to one class but to a group of classes. Conversely, when inspecting documents with high ensemble agreement (ie, cases in which soft labels approximate hard labels), we found clear pathology reports that focus on one specific class (Figure 5). The importance of this ensemble agreement and soft-label relationship is that we can guide the student model to make highly confident predictions for documents with little noise while lowering confidence for documents that involve multiple sites.

An additional benefit of ensemble model distillation is alleviating label noise. When predicting with 1000 models, one can naturally expect that a 100% agreement in predictions is likely to correspond with correct predictions. When inspecting pathology reports that were wrongly classified with 100% ensemble agreement, we found that these reports were actually incorrectly annotated (Figure 5). Consequently, ensemble model distillation is a useful tool to reduce label noise issues and is particularly beneficial in domains with a high number of highly related classes. This result is consistent with previous work that used ensembles for label correction.^35^^,^^36^

Notably, developing efficient abstention mechanisms is still an open area of research. In this study, we implemented softmax thresholding, which is a common abstention mechanism compatible with most DL models. We used the validation dataset to identify a threshold that would yield a 97% accuracy. However, we observed that when using this threshold in the holdout dataset, the resulting accuracy can be as low as 94.42% (see subsite in Table 4). We hypothesize that this performance disparity is amplified because of our leave-one-registry-out experimental setup, and it may be a sign of further overfitting. The discrepancy observed between the validation and test dataset highlights the need for future research that focuses on more efficient abstention techniques.

Our results yielded insight into the effect of ensemble size on model overconfidence. In this study, we implemented an ensemble of 1000 models. However, our results indicated that even an ensemble with between 4 and 100 models can reduce the number of wrong predictions made with high confidence. This highlights the accessibility of our methods because DL practitioners who have limited computational resources can still benefit from the overconfidence reduction obtained through ensemble model distillation.

The methods presented in this study are simple and highly parallelizable. The literature on model ensembles and distillation is extensive and includes a large variety of implementations. One can easily experiment by combining distillation with a particular ensemble technique such as boosting^37^ or bagging.^29^ In this paper, we focused on a simple implementation that involves training multiple versions of our current classifier with different initializations. Previous work has shown that the training models with distinct random seeds can infuse diversity in terms of the inductive biases learned by the networks.^38^ We hope that the simplicity of our method can provide a convenient solution for other DL practitioners who intend to reduce the number of highly confident but wrong predictions for the deployment of DL models in high-risk domains.

## CONCLUSION

Extreme class imbalance together with data and label noise leads to serious overfitting issues when training DL models for the classification of electronic cancer pathology reports. Ensemble methods are a simple solution to alleviate these issues, but these methods are computationally expensive and unsuitable for deployment by cancer registries across the country. Thus, this study quantified the use of ensemble model distillation as a low-resource alternative. The soft labels derived through model aggregation contain information about the variability in ensemble predictions. We showed that training a student model with the derived soft labels can reduce the number of highly confident but wrong predictions, thereby leading to a boost in abstention rates when using softmax thresholding. The implemented methods provide a simple and highly parallelizable solution for researchers working in high-risk domains. Our ensemble model distillation code is available on Github (https://github.com/kevindeangeli/EnsembleDistillation/).

## FUNDING

This manuscript has been authored by UT-Battelle LLC under Contract No. DE-AC05-00OR22725 with the US Department of Energy (DOE). The US government retains and the publisher, by accepting the article for publication, acknowledges that the US government retains a nonexclusive, paid-up, irrevocable, worldwide license to publish or reproduce the published form of the manuscript, or allow others to do so, for US government purposes. DOE will provide public access to these results of federally sponsored research in accordance with the DOE Public Access Plan (http://energy.gov/downloads/doe-public-access-plan).

This research was supported by the Exascale Computing Project (17-SC-20-SC), a collaborative effort of the DOE Office of Science and the National Nuclear Security Administration.

This research also used resources of the Oak Ridge Leadership Computing Facility at the Oak Ridge National Laboratory, which is supported by the DOE Office of Science under Contract No. DE-AC05-00OR22725.

This work has been supported in part by the Joint Design of Advanced Computing Solutions for Cancer program established by DOE and the National Cancer Institute (NCI) of the National Institutes of Health. This work was performed under the auspices of DOE by Argonne National Laboratory under Contract DE-AC02-06-CH11357, Lawrence Livermore National Laboratory under Contract DE-AC52-07NA27344, Los Alamos National Laboratory under Contract DE-AC5206NA25396, and Oak Ridge National Laboratory under Contract DE-AC05-00OR22725.

The collection of cancer incidence data used in this study was supported by the California Department of Public Health pursuant to California Health and Safety Code Section 103885; Centers for Disease Control and Preventions (CDCs) National Program of Cancer Registries (NPCR), under cooperative agreement 5NU58DP006344; the NCI Surveillance, Epidemiology and End Results (SEER) Program under contract HHSN261201800032I awarded to the University of California, San Francisco, contract HHSN261201800015I awarded to the University of Southern California, and contract HHSN261201800009I awarded to the Public Health Institute. The ideas and opinions expressed herein are those of the author(s) and do not necessarily reflect the opinions of the State of California, Department of Public Health, the NCI, the CDC, or their Contractors and Subcontractors.

KCR data were collected with funding from NCI’s SEER Program (HHSN261201800013I), the CDC’s NPCR (U58DP00003907), and the Commonwealth of Kentucky. KCR staff participation was also supported through the NCI Cancer Center Support Grant (P30 CA177558) support for the Markey Cancer Centers Cancer Research Informatics Shared Resource Facility. LTR data were collected using funding from NCI’s SEER program (HHSN261201800007I), the CDCs NPCR (NU58DP006332-02-00), and the State of Louisiana. NJSCR data were collected using funding from NCI’s SEER program (HHSN261201300021I), the CDCs NPCR (NU58DP006279), as well as the State of New Jersey and the Rutgers Cancer Institute of New Jersey. NMTRs participation in this project was supported by Contract HHSN261201800014I, Task Order HHSN26100001 from the NCI’s SEER program. The Cancer Surveillance System is supported by the NCIs SEER program (HHSN261291800004I) with additional funds provided by the Fred Hutchinson Cancer Research Center. UCR is funded by the NCI’s SEER program (HHSN261201800016I) and the CDC’s NPCR (NU58DP0063200) with additional support from the University of Utah and Huntsman Cancer Foundation.

## AUTHOR CONTRIBUTIONS

KD: investigation, methodology, software, visualization, and writing. SG, AB, and HY: conceptualization, investigation, methodology, writing, and supervision. ED, XW, AS, JD, SS, CW, LC, and LP: data curation and writing. GT: funding acquisition, supervision, and writing.

## CONFLICT OF INTEREST STATEMENT

None declared.

## Data Availability

The code for our implemented ensemble model distillation can be found at https://github.com/kevindeangeli/EnsembleDistillation/. The data underlying this article was provided by the state cancer registries by permission and cannot be shared publicly due to the privacy of individuals in the data corpus.
